# Contributory Role of Five Common Polymorphisms of *RAGE* and *APE1* Genes in Lung Cancer among Han Chinese

**DOI:** 10.1371/journal.pone.0069018

**Published:** 2013-07-11

**Authors:** Hongming Pan, Wenquan Niu, Lan He, Bin Wang, Jun Cao, Feng Zhao, Ying Liu, Shen Li, Huijian Wu

**Affiliations:** 1 School of Life Science and Biotechnology, Dalian University of Technology, Dalian, Liaoning, China; 2 Basic Medical Science College, Qiqihar Medical University, Qiqihar, Heilongjiang, China; 3 State Key Laboratory of Medical Genomics, Ruijin Hospital, Shanghai Jiao Tong University School of Medicine, Shanghai, China; Tor Vergata University of Rome, Italy

## Abstract

**Background:**

Lung cancer is the leading cause of cancer mortality in China. Given the ubiquitous nature of gene-to-gene interaction in lung carcinogenesis, we sought to evaluate five common polymorphisms from advanced glycosylation end product-specific receptor (*RAGE*) and apurinic/apyrimidinic endonuclease 1 (*APE1*) genes in association with lung cancer among Han Chinese.

**Methods and Results:**

819 patients with lung cancer and 803 cancer-free controls were recruited from Qiqihar city. Genotypes of five examined polymorphisms (*RAGE* gene: rs1800625, rs1800624, rs2070600; *APE1* gene: rs1760944, rs1130409) were determined by ligase detection reaction method. Data were analyzed by R software and multifactor dimensionality reduction (MDR). Hardy-Weinberg equilibrium was satisfied for all five polymorphisms. Overall differences in the genotype and allele distributions were significant for rs1800625 (P_genotype_<0.0005; P_allele_<0.0005), rs2070600 (P_genotype_ = 0.005; P_allele_ = 0.004) and rs1130409 (P_genotype_ = 0.009; P_allele_ = 0.004) polymorphisms. Haplotype C-A-A (alleles in order of rs1800625, rs1800624 and rs2070600) of *RAGE* gene was overrepresented in patients, and conferred a 2.1-fold increased risk of lung cancer (95% confidence interval: 1.52–2.91), independent of confounding factors. Further application of MDR method to five examined polymorphisms identified the overall best interaction model including rs2070600 and rs1130409 polymorphisms. This model had a maximal testing accuracy of 64.63% and a maximal cross-validation consistency of 9 out of 10 at the significant level of 0.006.

**Conclusions:**

Our findings demonstrated a potential interactive contribution of *RAGE* and *APE1* genes to the pathogenesis of lung cancer among Han Chinese. Further studies are warranted to confirm or refute these findings.

## Introduction

Lung cancer is the leading cause of cancer mortality in China, and its escalating prevalence presents a major public health challenge [1]. To unravel the genetic underpinnings of lung cancer, a proliferating range of single-locus investigations to genome-wide scans have been undertaken; however, neither a gene nor a variant hitherto has been confirmed uniformly across ethnic groups. One compelling reason might be attributable to the lack of consideration of gene-to-gene interaction, which is increasingly recognized as an ubiquitous component in the underlying etiology of most common diseases [2]. To shed some light on this issue, we, in this study, focused on two candidate genes, advanced glycosylation end product-specific receptor (*RAGE*) and apurinic/apyrimidinic endonuclease 1 (*APE1*), to explore their interactive association of common genetic defects with lung cancer risk.

Candidacy of *RAGE* and *APE1* genes for lung carcinogenesis is based on strong biological credentials [3]–[6]. Briefly, RAGE is a member of the immunoglobulin superfamily of cell surface molecules [7]. *In vivo* experiments suggested that both transcriptional and translational expression levels of RAGE were elevated in normal lung tissue, especially at the site of alveolar epithelium [8], but were inactivated in the corresponding tissue of non-small cell lung cancer patients [9]. RAGE was reported to impair the proliferative stimulus via fibroblasts in lung cancer cells [10], [11], supporting a role of RAGE in lung cancer progression. APE1 is a crucial enzyme in charge of the incision of DNA basic sites during base excision repair, and it functioned as a stimulator to the DNA binding activity of many transcription factors responsible for cancer promotion and progression. *In vitro* knock-down of *APE1* gene was observed to enhance the killing effect of hematoporphrphyrin derivative-mediated photodynamic therapy on non-small cell lung cancer cells [5]. As the genomic sequences of *RAGE* and *APE1* genes are highly polymorphic, it is of added interest to identify which genetic defect(s) might have functional potentials to affect the final bioavailability of these two genes, and thus to the pathogenesis of lung cancer.

To generate more information, we focused on five common polymorphisms from *RAGE* (rs1800625, rs1800624 and rs2070600) and *APE1* (rs1760944 and rs1130409) genes, and investigated their individual and interactive contribution to lung cancer risk among Han Chinese.

## Methods

### Study Population

All study participants were of Han Chinese descent, and resided in Qiqihar city, Heilongjiang province. This was a hospital-based case-control study encompassing 819 patients with lung cancer and 803 cancer-free controls. The institutional review board of Qiqihar Medical University approved this study, and each participant provided informed written consent at enrollment.

### Demographic Information

Data on age, gender, smoking, drinking, chronic obstructive pulmonary disease (COPD), and family history (within three generations) of cancer were recorded from each participant. COPD was diagnosed retrospectively. Smoking was defined as current smoking of at least one cigarette per day during the latest three months. Drinking was defined as having two or more standard drinks per week for men and one or more standard drinks per week for women during the latest three months.

### Diagnostic Information

The computed tomography (CT) scans were adopted to diagnose the presence of lung cancer, which was confirmed by senior respiratory physicians. When necessary, diagnosis was further confirmed by pathological biopsy. Participants with normal CT scan results and without family history of known cancers were treated as cancer-free controls. Lung cancer was clinically classified into squamous cell cancer, adenocarcinoma, and small cell cancer.

### Genotyping

Venous blood samples (2–5 mL) were collected in EDTA tubes for genomic DNA extraction (TIANamp Blood DNA Kit) and subsequent batch genotyping. Five examined polymorphisms were genotyped by the polymerase chain reaction-ligase detection reactions (PCR-LDR) method. Amplification parameters were 94°C for 2 min, 35 cycles of 94°C for 15 s, 60°C for 15 s, 72°C for 30 s, and a final extension step at 72°C for 5 min. Two specific probes and one common probe were synthesized for each polymorphism. The common probe was labeled at the 3′ end with 6-carboxy-fluorescein and phosphorylated at the 5′ end. The reacting conditions of LDR were 94°C for 2 min, 20 cycles of 94°C for 30 s and 60°C for 3 min. After reaction, 1 µL LDR reaction products were mixed with 1 µL ROX passive reference and 1 µL loading buffer, and then denatured at 95°C for 3 min and chilled rapidly in ice water. The fluorescent products of LDR were differentiated using ABI sequencer 377 (Applied Biosystems, USA).

### Statistical Analysis

Data were statistically analyzed with the use of the open-source R software (version 2.10) available at http://www.r-project.org and multifactor dimensionality reduction (MDR) (version 2.0) available at http://sourceforge.net/projects/mdr. A priori study power was estimated using PS (Power and Sample Size Calculations) software (version 3.0).

Unpaired t-test and χ^2^ test were used to compare continuous and categorical variables between patients and controls, respectively. Hardy-Weinberg equilibrium was evaluated by using a goodness-of-fit test. Logistic regression analyses were adopted under the assumptions of additive, dominant and recessive models for each polymorphism examined. Odds ratio (OR) and its corresponding 95% confidence interval (CI) were computed to quantify the association of genotypes with lung cancer risk. Statistical significance was set at P<0.05.

Haplotype frequencies and their risk prediction were calculated by Haplo.stats software developed by R software. In detail, haplo.em program was used to estimate frequencies; haplo.cc and haplo.glm programs were used to estimate OR and 95% CI according to a generalized linear model [12]. The differences in the estimated haplotype frequencies between patients and controls were based on simulated P-values. Simulated statistics are based on randomly permuting the trait and covariates and then computing the haplotype score statistics. The haplo.em, haplo.cc and haplo.glm were implemented using Haplo.stats software.

Gene-to-gene interactions were conducted by MDR method. MDR is a nonparametric (i.e., no hypothesis about the value of a statistical parameter is made) and model-free (i.e., assumes no particular inheritance model) data-mining alternative to classical logistic regression to detect and characterize nonlinear interactions among discrete variables [13], [14]. The general idea behind MDR method is that is reduces the dimensionality of the multilocus data by pooling the combinations of genotypes that can be defined as high risk and low risk according to the case-control ratio for the specific multilocus genotype [15]. In this study, all possible combinations of one to five polymorphisms were constructed, and a Bayes classifier in the context of 10-fold cross-validation was used to estimate the testing accuracy of each best model. A single best model had maximal testing accuracy and maximal cross-validation consistency, and the latter measured the number of times of 10 divisions of the data that the best model was found. Statistical significance was evaluated using a 1000-fold permutation test to compare observed testing accuracies with those expected under the null hypothesis of null association. Permutation testing corrected for multiple testing by repeating the entire analysis on 1000 datasets that were consistent with the null hypothesis.

## Results

### Baseline Characteristics

Baseline characteristics of the study population are summarized in Table 1. Distributions of age, gender and family history of cancers were comparable between patients with lung cancer and controls (P>0.05). Percentages of smokers (P<0.0005) and drinkers (P<0.0005), as well as history of COPD (P<0.0005), were remarkably higher in patients than in controls.

**Table 1:** The baseline characteristics of study population.

Characteristics	Patients (n = 819)	Controls (n = 803)	P**
Age (years)	57.35 (10.51)	57.04 (9.72)	0.846
Sex (male)	64.84%	64.76%	0.974
Smokers	36.26%	7.97%	<0.0005
Drinkers	16.85%	8.09%	<0.0005
COPD history	14.53%	4.73%	<0.0005
Family history of cancers	11.36%	10.83	0.738
Lung cancer subtypes			
Squamous cell cancer	36.16%	−*	
Adenocarcinoma	31.75%	−	
Small cell cancer	19.79%	−	
Others	12.3%		

Abbreviations: COPD, chronic obstructive pulmonary disease. Data are expressed as mean (standard deviation or SD) or percentage as indicated.

* data not available.

** P values were calculated by using unpaired t-test for age, and by χ2 test for other categorical characteristics.

### Single-locus Analysis

Genotype distributions and allele frequencies of five examined polymorphisms, as well as their risk prediction under various genetic models are presented in Table 2. No deviations from Hardy-Weinberg equilibrium were seen in both patients and controls for all polymorphisms. Overall, there were significant differences in the genotype and allele distributions of rs1800625 (P_genotype_<0.0005; P_allele_<0.0005), rs2070600 (P_genotype_ = 0.005; P_allele_ = 0.004) polymorphisms in *RAGE* gene and rs1130409 (P_genotype_ = 0.009; P_allele_ = 0.004) polymorphism in *APE1* gene, and the estimated study power to detect these differences was 94.2%, 81.6% and 81.3%, respectively.

**Table 2:** Genotype distributions and allele frequencies of five examined polymorphisms between lung cancer patients and controls, as well as the risk prediction under additive, dominant and recessive genetic models.

Polymorphism	Genotypeor allele	Patients (n = 819)	Controls (n = 803)	P_χ2_	Genetic models	OR; 95% CI; P	OR; 95% CI; P*
rs1800625-T/C	TT	447	485		Additive model	1.34; 1.14–1.57; <0.001	1.36; 1.16–1.59; <0.001
	CT	303	289	<0.0005	Dominant model	1.27; 1.04–1.55; 0.018	1.28; 1.05–1.56; 0.013
	CC	69	29		Recessive model	2.46; 1.57–3.83; <0.001	2.6; 1.67–4.04; <0.001
	C (%)	**26.92**	**21.61**	<0.0005			
rs1800624-T/A	TT	471	472		Additive model	1.08; 0.92–1.27; 0.326	1.1; 0.94–1.29; 0.226
	AT	289	287	0.411	Dominant model	1.05; 0.86–1.28; 0.604	1.07; 0.88–1.3; 0.528
	AA	59	44		Recessive model	1.34; 0.89–2.0; 0.156	1.42; 0.96–2.11; 0.081
	A (%)	**24.85**	**23.35**	0.319			
rs2070600-G/A	GG	321	352		Additive model	1.24; 1.07–1.44; 0.004	1.25; 1.08–1.45; 0.003
	AG	382	377	0.005	Dominant model	1.21; 0.99–1.48; 0.058	1.22; 1.0–1.48; 0.053
	AA	116	74		Recessive model	1.63; 1.19–2.22; 0.002	1.66; 1.22–2.27; 0.001
	A (%)	**37.48**	**32.69**	0.004			
rs1760944-G/T	GG	321	336		Additive model	1.1; 0.95–1.27; 0.195	1.1; 0.95–1.26; 0.213
	GT	384	369	0.429	Dominant model	1.12; 0.92–1.36; 0.277	1.11; 0.91–1.36; 0.287
	TT	114	98		Recessive model	1.16; 0.87–1.55; 0.306	1.16; 0.86–1.54; 0.338
	T (%)	**37.36**	**35.18**	0.196			
rs1130409-G/T	GG	498	531		Additive model	1.28; 1.08–1.51; 0.005	1.3; 1.1–1.54; 0.002
	GT	273	247	0.009	Dominant model	1.26; 1.03–1.54; 0.026	1.27; 1.04–1.56; 0.019
	TT	48	25		Recessive model	1.94; 1.18–3.17; 0.009	2.1; 1.29–3.42; 0.003
	T (%)	**22.53**	**18.49**	0.004			

*Abbreviations*: OR, odds ratio; 95% CI, 95% confidence interval.

* P values were adjusted for age, gender, smoking and drinking.

P_χ2_ was calculated by χ^2^ test for differences in genotypes and alleles between patients and controls.

Across all genetic models, carriers of mutant allele or genotype of polymorphisms rs1800625 and rs1130409 polymorphisms were significantly associated with lung cancer risk, especially under the recessive model, even after adjusting for confounding factors. With regard to rs2070600 polymorphism, significance was merely attained under additive and recessive models.

### Haplotype Analysis

Given that *RAGE* and *APE1* genes are mapped to different chromosomes, haplotype analyses were conducted separately for each gene. Haplotype frequencies and their risk prediction for lung cancer are presented in Table 3. Frequencies of the most common haplotype in both *RAGE* (T-T-G in order of rs1800625, rs1800624, and rs2070600, P_sim_ = 0.315) and *APE1* (G-G in order of rs1760944 and rs1130409, P_sim_ = 0.084) genes were similar between patients and controls. Compared with controls, haplotype C-A-A in *RAGE* gene was overrepresented in patients (Study power: 99.5%), and was associated with a 2.1-fold increased risk of lung cancer (95% CI: 1.52–2.91) before adjustment and a 2.15-fold increased risk after adjustment (95% CI: 1.55–2.97). There were no significant differences in the haplotype frequencies of *APE1* gene between two groups.

**Table 3:** Haplotype frequencies of examined polymorphisms between lung cancer patients and controls, as well as their risk prediction.

Haplotype	Patients	Controls	P_sim_	OR; 95% CI	OR; 95% CI^*^
***RAGE*** ** gene (in order of rs1800625–rs1800624–rs2070600)**					
T-T-G	30.23%	32.95%	0.315	Reference group	Reference group
T-A-G	11.74%	11.96%	0.855	1.01; 0.79–1.29	1.04; 0.82–1.32
C-T-G	17.35%	19.15%	0.259	0.99; 0.8–1.21	0.98; 0.79–1.19
C-A-G	10.19%	9.11%	0.334	1.22; 0.95–1.58	1.24; 0.96–1.59
C-A-A	7.72%	3.99%	0.009	2.1; 1.52–2.91	2.15; 1.55–2.97
T-T-A	13.12%	12.55%	0.681	1.14; 0.91–1.43	1.12; 0.89–1.41
C-T-A	7.06%	6.0%	0.259	1.29; 0.96–1.74	1.31; 0.97–1.76
***APE1*** ** gene (in order of rs1760944–rs1130409)**					
G-G	43.24%	45.87%	0.084	Reference group	Reference group
T-G	28.53%	25.94%	0.195	1.16; 0.98–1.37	1.17; 0.99–1.38
G-T	18.81%	20.17%	0.249	0.95; 0.79–1.15	0.97; 0.81–1.17
T-T	9.42%	8.02%	0.568	1.38; 1.06–1.79	1.34; 1.03–1.75

*Abbreviations*: OR, odds ratio; 95% CI, 95% confidence interval. Psim: simulated P-value, which was calculated based on randomly permuting the trait and covariates and then computing the haplotype score statistics.

### Gene-to-gene Interaction Analysis

An exhaustive MDR analysis on the possible interaction of five examined polymorphisms is summarized in Table 4. Each best model was accompanied with its testing accuracy, cross-validation consistency and significant level determined by permutation testing. The overall best MDR model encompassed polymorphism rs2070600 in *RAGE* gene and rs1130409 in *APE1* gene. This model had a maximal testing accuracy of 64.63% and a maximal cross-validation consistency of 9 out of 10 at the significant level of 0.006.

**Table 4:** Summary of MDR analysis.

Best combination of each model	Testing accuracy	Cross-validation consistency	P
rs1130409	0.5961	8	0.174
rs2070600, rs1130409	0.6563	9	0.006*
rs1800625, rs2070600, rs1130409	0.6329	7	0.101
rs1800625, rs1800624, rs2070600, rs1130409	0.6257	7	0.213
rs1800625, rs1800624, rs2070600, rs1130409, rs1760944	0.6097	10	0.304

* The overall best MDR model.

## Discussion

In this study, we sought to investigate the association of five common polymorphisms from two candidate genes with lung cancer risk in a large Han Chinese population involving 1622 individuals. The most noteworthy finding was that genetic interaction between *RAGE* and *APE1* genes might confer a potentially increased risk for lung cancer, which was reinforced by the results of single-locus and haplotype analyses. To the authors’ knowledge, this study represents the first so far to explore the potential interaction between *RAGE* and *APE1* genetic polymorphisms in predisposition to lung cancer.

In view of potential biological candidacy, the mechanisms for the involvement of RAGE and APE1 in lung carcinogenesis remains to be elucidated. A literature search revealed little evidence on the association of *RAGE* gene polymorphisms with lung cancer. In a previous study by Schenk et al [9], a promoter polymorphism (T-388A) in *RAGE* gene was reported to be a putative risk locus for the development of non-small cell lung cancer. Extending this observation, we, in a large Han Chinese population, examined three common polymorphisms in *RAGE* gene and found that carriers of mutant genotypes of promoter polymorphism rs1800625 and coding polymorphism rs2070600 in 3rd exon exhibited strikingly increased risk for lung cancer, which was further potentiated by our following haplotype analyses. More recently, a systematic review of 3491 lung cancer patients and 4708 controls detected significant association of Asp148Glu (rs1130409) polymorphism with lung cancer, especially in Asian populations [16], in agreement with the results of our single-locus analyses. In this context, it is reasonable to hypothesize that genetic defects of *RAGE* and *APE1* genes might increase the risk of developing lung cancer.

Although the candidate gene approach cannot replace the genome-wide scan strategy in unraveling the genetic architecture of complex diseases, it is an essential alternative strategy [17], particularly when the selection of candidate genes is biologically sound, the recruited population is relatively large and homogeneous, and the analytical methods are solid. As recommended, recruitment of 1000 individuals or more in each group is required to yield a firm conclusion [18]. Despite our sample sizes encompassing 819 patients and 803 controls does not give us this capability, given the wide differences of genetic distributions, a priori power calculation suggested that we had more than 80% power to detect the significant polymorphisms or haplotypes of realistic effect sizes. Notably in this study, all subjects were ethnically homogeneous and enrolled from Heilongjiang province, where the prevalence of lung cancer is relatively high due to the indoor air pollution from the unventilated coal-fueled stoves. Moreover, there were no deviations from the Hardy-Weinberg equilibrium for all examined polymorphisms, excluding the possibility of biased results by faulty genotyping or population stratification [19]. Further data from 1622 study participants were analyzed with statistical consideration of traditional confounders. Although residual confounding by incompletely measured or unmeasured physiologic covariates might exist, it seems unlikely that our findings might be interpreted by confounding.

To enhance the likelihood of identifying disease-causing genetic defects, we employed a promising data-mining analytical approach, MDR, which is nonparametric and genetic model-free nature in design [20]. Considering the ubiquity of genetic interactions in the pathogenesis of complex diseases, the identification and characterization of susceptible genes or variants require a thorough understanding of gene-to-gene interaction [21]. Using MDR model, we teased out two polymorphisms respectively from *RAGE* and *APE1* genes with strong interactive effect, reinforcing the results of our single-locus and haplotype analyses, and lending support for gene-to-gene interaction in the development of lung cancer. Therefore, MDR method might represent the first step in providing clues to guide further research.

Interpretation of our results, however, should be viewed in light of several limitations. First, this study was retrospective in design, which precludes further comments on the cause-effect relationship [22]. Second, we only focused on five common polymorphisms, and is encouraged to examine more polymorphisms, especially the low-penetrance polymorphisms from other promising cancer-susceptibility genes, such as *PTGS2* and *CYP2E1* genes [23]. More importantly, because lung cancer is a multifactorial disease [24], characterizing the interaction of polymorphisms from different chromosomes is regarded as an effective approach to elucidate its genetic architecture. Third, the MDR method used in this study has some underling drawbacks including computational intensiveness, indistinct interpretation, lack of sensitivity, and heterogeneity-free assumption [20], [25]. Fourth, the fact that our study participants were of Han Chinese ancestry limited the generalizability of our findings, calling for further confirmation in other ethnic groups.

Despite these limitations, our results collectively demonstrated a potential interactive contribution of *RAGE* and *APE1* genes to the pathogenesis of lung cancer among Han Chinese. Nevertheless, for practical reasons, we hope that this study will not remain just another endpoint of research instead of a beginning to establish background data to further investigate the molecular mechanisms of *RAGE* and *APE1* genes in lung carcinogenesis.
